# Targeting CD24/Siglec-10 signal pathway for cancer immunotherapy: recent advances and future directions

**DOI:** 10.1007/s00262-023-03606-0

**Published:** 2024-01-27

**Authors:** Xingchen Li, Wenzhi Tian, Zhongxing Jiang, Yongping Song, Xiangyang Leng, Jifeng Yu

**Affiliations:** 1 The First Affiliated Hospital of Zhengzhou University, Zhengzhou, 450052 China; 2 Affiliated Hospital of Changchun University of Traditional Chinese Medicine, Changchun, 130021 Jilin China; 3 ImmuneOnco Biopharmaceuticals (Shanghai) Inc., Shanghai, 201203 China; 4 Henan International Joint Laboratory of Nuclear Protein Gene Regulation, Henan University College of Medicine, Kaifeng, 475004 Henan China

**Keywords:** CD24, Siglec-10, Signaling pathway, Monoclonal antibody, Cancer immunotherapy

## Abstract

The small, heavily glycosylated protein CD24 is primarily expressed by many immune cells and is highly expressed mostly in cancer cells. As one of the most crucial biomarkers of cancers, CD24 is frequently highly expressed in solid tumors, while tumor-associated macrophages express Siglec-10 at high levels, Siglec-10 and CD24 can interact on innate immune cells to lessen inflammatory responses to a variety of disorders. Inhibiting inflammation brought on by SHP-1 and/or SHP-2 phosphatases as well as cell phagocytosis by macrophages, the binding of CD24 to Siglec-10 can prevent toll-like receptor-mediated inflammation. Targeted immunotherapy with immune checkpoint inhibitors (ICI) has lately gained popularity as one of the best ways to treat different tumors. CD24 is a prominent innate immune checkpoint that may be a useful target for cancer immunotherapy. In recent years, numerous CD24/Siglec-10-related research studies have made tremendous progress. This study discusses the characteristics and workings of CD24/Siglec-10-targeted immunotherapy and offers a summary of current advances in CD24/Siglec-10-related immunotherapy research for cancer. We then suggested potential directions for CD24-targeted immunotherapy, basing our speculation mostly on the results of recent preclinical and clinical trials.

## Introduction

With immune checkpoint inhibitors (ICIs), novel targeted immunotherapy has gained popularity as a cancer therapeutic strategy and has recently demonstrated potent antitumor activity in a range of malignancies. Examples of ICIs that target several signaling pathways include cytotoxic T-lymphocyte-associated protein 4 (CTLA-4), programmed cell death protein 1 (PD-1)/programmed death ligand 1 (PD-L1), and others [1, 2]. Medical oncology's therapy landscape has changed as a result of CD47/signal regulatory protein *α* (SIRP *α*) improving patient outcomes [3, 4]. The main rationale for suppressing an immune response is the regulation of antitumor T cells by a number of immunological checkpoints. Immune checkpoint treatment results in long-lasting therapeutic effects by restoring the induction, activation, and proliferation of tumor-specific cytotoxic T cells. ICIs targeting PD-1/PD-L1, CTLA-4, lymphocyte activating gene 3 (LAG3), and CD47/SIRP- have been approved by the FDA for use in a range of solid tumors and hematological malignancies. Despite most patients with tumor responses have long-term illness control, one-third of patients experience relapse. Acquired resistance mechanisms present a significant difficulty because they are currently not well understood. With the help of new generations of immunological checkpoint inhibitors and new ICIs targeting various signaling pathways, resistance mechanisms may be overcome. Following the discovery of the PD-1/PD-L1, CTLA-4, LAG3, and CD47/SIRPα signaling pathways, a new checkpoint of cluster of differentiation 24 (CD24)/Siglec-10 (sialic acid-binding immunoglobulin-like lectin-10) signaling route has been identified. The CD24/Siglec-10 axis can avoid a fatal reaction including pathological cell death [3].

Targeting the CD24/Siglec-10 axis has emerged as a viable immunotherapy strategy based on the findings of current preclinical studies and clinical trial outcomes. In two clinical trials, monoclonal antibodies (mAbs) against CD24 were proven to be both clinically safe and tolerated [4, 5]. It has been investigated how the recombinant fusion protein CD24Fc, which activates the CD24/Siglec-10 pathway, affects patients with advanced solid tumors including melanoma [6]. This review addresses the properties and mechanisms of CD24/Siglec-10-targeted immunotherapy and provides an overview of recent developments in CD24/Siglec-10-related research in cancer immunotherapy. Then, speculating primarily on the findings of recent preclinical and clinical trials, we proposed potential avenues for CD24-targeted immunotherapy.

## CD24/Siglec-10: structures, expression, function, and the signaling pathways

### The structures of CD24/Siglec-10

CD24, a small and heavily glycosylated protein, was formerly known as heat-stable antigen (HSA). It consists of 31–34 amino acids held to the cell surface by a glycosylphosphatidylinositol anchor [6]. The gene for mouse CD24, located on chromosome 6q21, was discovered more than 40 years ago and codes for a glycosylated protein with 16 possible O- and N-glycosylation sites [7]. The mature peptide backbone of CD24 had four possible N-linked and multiple O-linked glycosylation sites [8]. The molecular weight of CD24 ranges from 35 to 45 kDa, and its glycosylation modifications change significantly between various cell types [8, 9]. The human CD24 molecule has additional serine and threonine residues, rendering the molecule similar to a mucin [10]. Additionally, the weight of the CD24 molecule can be artificially altered due to its co-migration with IgG and IgM heavy chains, respectively [7, 11].

Siglecs, often referred to as sialic acid-binding immunoglobulin-like lectins, are type I Ig-like transmembrane protein receptors. They have IgV-like domains that bind to sialic acid linked to the terminal portions of cell surfaces expressed by various immune cells [12–14]. Siglecs are split into two groups based on their structural similarity. Siglec-1, 2, 4, and 15 make up the first group. They share between 25 and 30% of their amino acid composition with humans, rats, and other vertebrates. The second category of Siglecs consists of Siglec-3/CD33 and CD33-related Siglecs, which have less vertebrate-specific conservative structures but significant levels of extracellular domain homology (50–85% amino acid identity) with CD33. The Siglecs that are connected to CD33 in humans are Siglec-3, 5, 6, 7, 8, 9, 10, 11, 12, 14, and 16, while the Siglecs that are associated with CD33 in mice are Siglec-3, -E, F, G, and H [15]. CD24 can bind to Siglec-10, -5 and E-/P- selectins [16], but not to other Siglecs, including Siglec-7 and -11. Siglec-10 forms a complex with high-mobility group box-1 through CD24. Despite the fact that all Siglecs are capable of identifying sialoglycans, these receptors' binding preferences differ greatly [17]. Siglec-10 preferably recognizes sialic acid ligands with *α* 2–3- or 2–6-linkage. When binding to receptors, the distinct structure of the complete CD24 molecule can attract Siglec-10 [18]. However, it is still explored whether the protein–protein interaction and/or the density of glycosylation on CD24 are involved in making CD24 specific for Siglec-10 over all the other Siglecs. Importantly, targeted mutation of CD24 significantly reduced Siglec-10 binding to mouse spleen cells, which suggests that CD24 is likely the dominant ligand for Siglec-10 on the hematopoietic cells [3]. It has been discovered that a number of cancers highly express sialylated glycans, which bind to Siglec-10 and use the CD24/Siglec-10 interaction to evade the immune system around the tumor [19]. Furthermore, soluble CD52 molecules will interact with the inhibitory Siglec10 to significantly inhibit T-cell proliferation and activation [20]. CD24 has multiple ligands that interact either in cis (on the same cell) or in trans (on a different cell), including P-, L-, and E-selectin, high-mobility group box 1 (HMGB1), L1 cell adhesion molecule (L1CAM), neural cell adhesion molecule (NCAM), and Siglec-G [21], while Siglec-10 is the main ligand for CD24 [22].

### Expressions of CD24 and Siglec-10

CD24, a glycosylphosphatidylinositol (GPI)-anchored protein, is expressed on the membranes of cancerous cells, as well as in the cytoplasm and nuclei of some cancerous cells, such as tissue and tumor stem cells [3]. In addition to these cell types, CD24 is primarily expressed in many immune cells, including B and T lymphocytes, monocytes, granulocytes, epithelial cells, neural cells, and muscle cells [23]. It is commonly utilized as a marker for hematopoietic and neuronal cell differentiation [1, 3]. FoxP3^+^ regulatory T cells also express CD24, which plays a role in the regulation and differentiation of CD8 + T cells [22, 24]. More significantly, CD24 has been regarded as a cancer biomarker since cancer cells frequently express it, particularly in solid tumors [25, 26]. In addition to CD44, CD24 can also be used to identify the cancer stem cells (CSCs) [27]. Elevated CD24 gene expression is seen in those with non-alcoholic fatty liver disease (NAFLD), and this is thought to be a risk factor. Furthermore, compared to patients with fibrosis stage F0, patients with fibrosis stage F1 express more CD24 [28].

An in-depth analysis of the competitive endogenous RNA (ceRNA) network between CD24-high and CD24-low tumor samples of breast carcinoma, using transcriptome profiles from the TCGA database, identified the CD24-associated ceRNA RP1-228H13.5/miR-135a-5p/BEND3 and SIM2 axis as potential therapeutic targets and predictors for breast invasive carcinoma (BRCA) diagnosis and prognosis [24].

Patients with triple-negative breast cancer (TNBC) and ovarian cancer have a fraction of tumor-associated macrophages (TAM) in the tumor microenvironment (TME) that highly express Siglec-10, the main ligand for CD24, which is widely expressed by B cells, activated T cells, and monocytes [22]. Unstimulated macrophages generated from human donors can strongly express Siglec-10 in response to the inhibitory cytokines TGF-1 and IL-10 [24]. Siglec-10 is highly expressed in M2-like macrophages produced by immunosuppressive drugs. Many tumors highly express CD24 and that TAMs express high levels of Siglec-10. Both genetic ablation of CD24 or Siglec-10, and mAb blockade of the CD24–Siglec-10 interaction, significantly increase the phagocytosis of all CD24-expressing human tumors tested [29]. Through highly expression of CD24, tumor cells interact with Siglec10, which inhibits the activity of macrophages and achieves immune escape [6, 29]. The CD24/Siglec-10 complex engages danger-associated molecular pattern (DAMP) molecules [3, 24] and initiates the SHP-1 and/or SHP-2 phosphatases-related signaling cascade [3, 22, 24].

### CD24/Siglec-10 axis and its functions

Siglec-10, Siglec E, P-selectin, and L1-cell adhesion molecule are among the proteins that CD24 binds to, enabling it to perform a variety of activities. To prevent phagocytosis, the failure of macrophages' phagocytic function is solely connected with CD24 binding to Siglec10. In addition to binding to Siglec-10 on innate immune cells, CD24 also inhibits inflammatory responses in a variety of illnesses, including infection [30], sepsis [26], liver injury [3], and chronic graft-versus-host disease (GVHD) [28]. CD24 binding to Siglec-10 can activate SHP-1 and/or SHP-2 phosphatases linked to tyrosine-based inhibitory motifs (ITIMs), which prevents TLR-mediated inflammation and macrophage engulfment of cells [14, 26, 30]. CD24/Siglec-10 binding has also associated with autoimmune disorders, GVHD, and tumor progression [28, 29, 31].

The roles of cytoplasmic CD24 in tumor growth and metastasis are not fully understood. Intracellular CD24 can build up in the cytoplasm as a result of GPI system flaws [6], which can hinder the growth of tumor cells [30] by weakening and inactivating p53[26]. The CD24-p53 axis inhibits the development of cancer by preserving intrahepatic macrophages. P53 keeps the intrahepatic macrophage activity up in hepatocellular carcinoma (HCC), which aids in the removal of DNA-damaged hepatocytes [30]. While one study found that CD24 increases cell invasion by enhancing contractility and encouraging cell adherence to fibronectin and collagen I and IV [32], another experiment discovered that intracellular CD24 inhibits tumor cell invasion and metastasis by affecting the posttranscriptional regulation of binder of arl two (BART) through G3BP RNase activity [30].

A different study discovered that the genes GATA3, CD24, and Siglec-10 are substantially expressed in the tissues and cells of ovarian cancer (OC). Siglec-10's expression was increased by GATA3 upregulating CD24. The in vivo assay proved that the activation of the CD24/Siglec-10 axis by GATA3 provided by TAM-derived extracellular vesicles (EVs) was successful in inducing OC. TAM-derived EVs containing GATA3 increase tumor development and OC cell resistance to therapy via the CD24/Siglec-10 axis [30].

According to studies using a mouse model, TNBC cell production of CD24 promotes the effective priming of T lymphocytes in lymph nodes [33]. Due to limited adoptive CD4^+^ T-cell growth and rapid lymph node cell death, CD24-deficient mice show poor T-cell priming. The host immune responses against CD24 by NK, T, and B lymphocytes did not contribute to the poor growth of T cells. T-cell concentration and survival in draining lymph nodes were restored in CD24-/- mice by transgenically expressing CD24 on DC. It was demonstrated that a polyclonal T-cell response to an antigen was similarly reduced in the lymph nodes of CD24-/- animals by utilizing MHC II tetramer tagging [34]. These findings imply that CD24 inhibition may reduce unwanted T-cell responses, such as those observed in autoimmune disorders [30].

### The regulation of CD24

The Siglecs are a group of sialic acid-binding, immunoglobulin-like lectins that are thought to improve cell–cell communication and regulate immune system cell behavior by identifying glycans [14]. The Siglecs feature ITIM or ITIM-like motifs in cells and are able to detect the N-terminal of ligands [35]. A large number of these motifs are associated with protein tyrosine that have an SH2 domain, such as phosphatase 1 (SHP-1) and SHP-2 [14]. Siglecs bind to the sialic acid linked to the glycoconjugates on the cell surface upon recognition of the sialic acid-containing structure [14].

Siglecs' recognition of ligands leads to induction of cytoplasmic ITIM or ITIM-like tyrosine accessibility to Src family kinases such as Lyn. These Src family kinases phosphorylate the ITIM domain after Siglec activation. As a result of phosphorylation, cellular activation is subsequently inhibited and SHP-1 and/or SHP-2 phosphatases with SH2-domains are then recruited to further weaken signal transduction [12, 15, 35, 36]. Mouse Siglec-G is the homolog of human Siglec-10 [15]. It has been demonstrated that Siglec-G associates with *Cbl* through a CD24-independent method to degrade the retinoic acid-inducible gene 1 and decrease the generation of type I interferon in response to RNA virus infection. The host may be able to distinguish between infectious nonself and nonself through the negative regulation of Siglec-G/10 [15]. Siglec-G on T cells must interact with CD24 to suppress DAMP-mediated amplification of their responses. In the B6-BALB/c paradigm, the mortality of allogeneic CD24-/- mice was noticeably higher than that of allogeneic wild-type controls. By joining the extracellular domain of mature human CD24 with the Fc domain of human immunoglobulin G1 (IgG1), a novel CD24Fc fusion protein can improve the interaction between CD24 and Siglec-G as a direct agonist. CD24Fc treatment significantly reduced graft vs host disease in B6 wild-type animals [31]. Recently, CD24 has been regarded as one of the most effective treatments for a variety of malignancies and has the potential to be the dominant innate immune checkpoint [4, 26, 28, 29, 37].

Proteins involved in GPI assembly and N and O glycosylation, including as PGAP2, PIGP, and PIGN, control the membrane bound CD24 protein. Numerous factors control CD24 in different type of tumors, according to studies. In human bladder cancer, urothelial carcinoma, and breast cancer, respectively, CD24 is increased by hypoxia-inducible factor 1alpha (HIF1*α*), androgen receptor and DNA methyltransferase, estrogen receptor, and truncated glioma-associated oncogene homolog 1 [32, 34, 37, 38]. On the other hand, CD24 expression is downregulated by Twist, -catenin/TCF, miR-34a and miR-146a, and histone deacetylase (HDAC) in breast cancer, colorectal, oral squamous cell carcinoma, and HDAC-positive colorectal cancer, respectively [34, 38–41]. According to another study, SIM2 upregulates CD24 and cytokeratin 4 (CK4), which are prognostic indicators for chemoradiotherapy and surgery in esophageal cancer [42]. After chemotherapy and radiation therapy, a positive prognosis was linked to high CD24 and KRT4 mRNA expression [42].

Mutant p53 can be protected from degradation by the ARF-NPM interaction, but tumor cell cytoplasmic CD24 can interfere with this contact, inactivating and destabilize p53 [43]. One study found that intracellular CD24 increases tumor cell invasion by encouraging cell contractility and encouraging cell adhesion to fibronectin and collagen I and IV [44]. A further investigation, however, revealed that intracellular CD24 inhibits tumor cell invasion and metastasis by impacting the posttranscriptional control of BART via G3BP RNase activity [45].

A versatile protein called calreticulin (CRT) is present in the ER. CRT contributes to the development of tumors and encourages the growth and migration of malignant cells. The CRT is transported to the cell surface when cancer cells experience immunogenic cell death and acts as a "eat me" signal, encouraging dendritic cells to phagocytose the tumor cells and enhancing the sensitivity of malignancies to anticancer immunotherapy. Strong cancer immunotherapy effects can be achieved by simultaneously inhibiting CD24 and activating CRT in macrophages [46]. Consequently, CRT is viewed as a cancer treatment target as well as a diagnostic marker [46].

ADP-ribosylation of the RNA helicase DDX5 by poly (ADP-ribose) polymerase 1 (PARP1) controls CD24 transcription in pancreatic cancer, according to research. It was shown that the innate immunosuppressive factor CD24 was increased when PARP1 was suppressed. Targeting CD24 had a confirmed activating effect on macrophage phagocytosis. PARP1 ADP ribosylated the transcription factor DDX5, which decreased the transcription of CD24. Pancreatic cancer patients who received combined PARP1 and CD24 inhibition experienced a synergistic antitumor effect [47]. Polymorphic STOX1-A/B gene variations can suppress CD24 in trophoblast cell lines, according to an in vitro investigation that modeled preeclampsia [48].

### Preclinical trials targeting CD24/Siglec-10

Preclinical research has demonstrated the promise of CD24 inhibition as a targeted cancer immunotherapy for several malignancies. When it comes to ovarian and breast malignancies, CD24 may be the major innate immune checkpoint. Research on the function of the CD24/Siglec-10 axis in controlling macrophage-triggered phagocytosis using human breast cancer MCF-7 cells has demonstrated that in an in vitro co-culture system, macrophages preferentially phagocytotize CD24-/-MCF-7 cells over wild-type MCF-7 cells [29]. The phagocytosis of the MCF-7 cells in their wild-type state was markedly improved by Siglec-100-/-macrophages. The phagocytosis of MCF-7 cells in their wild-type state by macrophages was significantly enhanced by anti-CD24 antibody treatment [29].

Further evidence that the CD24/SIGLEC-10 axis functions as a "don't eat me" signal in tumor immunity comes from the finding that inhibition of the CD24/SIGLEC-10 axis can increase phagocytosis more than CD47 blocking therapy [29]. MCF-7 cells that were CD24-knockout had a significantly lower tumor burden than the wild-type group, according to in vivo xenograft model studies [29]. Furthermore, when macrophages were depleted, CD24 knockout animals but not wild-type mice displayed a significant reduction of the decrease in tumor load, suggesting that the antitumor effect may be brought on by macrophage-mediated phagocytosis [29]. These results suggest that the combined blockade of CD24 and CD47 may have clinical potential in cancer immunotherapy because the CD24/Siglec-10 axis can regulate macrophage-mediated phagocytosis.

The CD24/Siglec-10 axis is another possible immunotherapeutic target for mantle cell lymphoma (MCL). Aroldi et al. demonstrated that CD24 mAb improved macrophage phagocytosis using a phagocytic assay via co-culture of M2-like macrophages with MCL cell lines [49]. Another in vitro investigation discovered that CD24 mAb induced autologous macrophages to phagocytose primary patient-derived MCL cells and eliminated more than 90% of MCL cells [50].

Treatment for MCL using CD24 mAb was superior to CD47 mAb, suggesting that CD24 mAb may be more effective in treating MCL than CD47 mAb [49]. Contrarily, in diffuse large B-cell lymphoma (DLBCL), CD24 mAb treatment was less successful than CD47 mAb treatment [49]. High mRNA expression of CD24 linked with poor overall survival in MCL and follicular lymphoma patients, while CD47 expression did not [49]. The phagocytic clearance of CD24-positive MCL cell lines and primary autologous MCL blasts was improved in vitro by CD24 mAb treatment. In MCL but not in DLBCL, CD24 mAb treatment had a stronger effect than CD47 InhibRx treatment. Low overall survival was associated with high CD24 expression in MCL, but not in DLBCL. Consequently, CD24 mAb therapy may provide as an alternate therapeutic strategy for MCL [49]. Another study found that individuals with esophageal cancer who had high CD24 mRNA expression had a better outcome after chemoradiotherapy. High CD24 and CK4 protein expression were found to be independent predictors of a favorable outcome in response to chemoradiotherapy by multivariate analysis [42].

Epithelial circulating tumor cells (CTCs) were shown to be significantly correlated with ER expression (*p* = 0.036) and TNM stage (*p* = 0.018) in a recent study on the expression of CD24 in peripheral blood CTCs and the utility of CTCs in predicting the prognosis of breast cancer patients. Breast cancer patients' mixed epithelial/mesenchymal-CTCs were significantly associated with lymph node metastases (*p* = 0.026). In CTCs, CD24 was expressed positively at a rate of 58.82% (60/102). TNM stage, lymph node metastasis, and tumor size all had a strong correlation with the number of CD24-positive CTCs (*p* = 0.002, 0.020, and 0.025, respectively). Therefore, in breast cancer patients, the TNM stage, lymph node metastasis, and tumor size are all closely connected to the positive expression of CD24 in CTC. One predictive predictor for individuals with early and intermediate-stage breast cancer is the positive expression of CD24 in CTCs, particularly in mixed-CTCs [51, 52].

By combining nanospheres and CD24 antibodies to create a unique antiCD24 nanosphere, the CD24/Siglec-10 signaling pathway is blocked, which regulates CD24 degradation and partially recovers the macrophages' capacity to phagocytose tumor cells. In addition to successfully restoring macrophage function in vitro, the combination of nanosphere-antiCD24 with glucose oxidase, an enzyme that facilitates the oxidative breakdown of glucose, reduces tumor growth in xenograft animal models with no discernible harm to healthy tissues. The results suggest that nanosphere-antiCD24 may be used in tumor therapy to disrupt the CD24/Siglec-10 signaling pathway and degrade membrane proteins [53].

In TNBC, the CD24/Siglec-10 signaling pathway has emerged as a fresh and powerful immunological checkpoint. According to a recent study {Zhao, 2023 #8}, engineered nanoparticles (P-aCD24/CEL + P/shMFN1) have tremendous potential for treating TNBC because they enhance CD24 blockage and mitochondrial dynamics modulation in TNBC therapy. To transport anti-CD24 mAb (aCD24), celastrol (CEL), and mitofusin 1 shRNA (shMFN1) for synergistic tumor cell-targeted therapy and TAM-targeted immunomodulation, engineering nanoparticles were created [31, 33, 51].

The combined response of the carrier to pH and MMP2 in the tumor microenvironment allowed P-aCD24/CEL to successfully release aCD24. CEL reactivated macrophage phagocytosis of tumor cells, improved macrophage-based immunotherapy, and induced immunogenic cell death of tumor cells. These effects were accompanied by a decrease in the "don't eat me" signal CD24 and an increase in the "eat me" signal CRT [31].

Epidermal growth factor receptor (EGFR) can help cells grow and divide. A mutation, or damage, in an EGFR gene causes the tumorgenesis, such as in EGFR-positive non-small cell lung cancer (NSCLC) patients. EGFR is an important biomarker identified as a potential “target” for personalized treatments in lung cancer. When EGFR-tyrosine kinase inhibitors (TKI)-treated EGFR-mutant cells were cultured in vitro with anti-CD24 antibodies, monocyte-derived macrophages promoted antibody-dependent cell phagocytosis (ADCP), indicating that CD24 may be a therapeutic target for EGFR-mutant lung cancer. Additionally, EGFR inhibition sped up the release of cell-free DNA (cfDNA) from senescent tumor cells. Furthermore, EGFR inhibition in NSCLC cells with EGFR mutations promotes a tumor microenvironment linked to immune evasion. Therefore, CD24-targeted therapy with cfDNA monitoring may help patients with EGFR-mutant NSCLC achieve better treatment results [53].

It has been reported that CD24 expression has a correlation with a poor prognosis in different disease types [54, 55]. For example, the study conducted in pancreatic cancer revealed that surface CD24 may play a role in the inhibition of cell invasion and metastasis and that intracellular CD24 inhibits invasiveness and metastasis through its influence on the posttranscriptional regulation of BART mRNA levels via G3BP RNase activity [45]. Its expression in tumor stem cells can induce tumor resistance to chemoradiation and promote and tumor recurrence, therefore with a significant correlation with poor survival [55]. Further investigations need to be done to explore the clinical significance of CD24 expression in relation to different cancers.

### Clinical trials targeting CD24/Siglec-10

Only a few clinical trials have been started, despite the fact that much preclinical research focusing on the CD24/Siglec-10 signaling pathway have been conducted. After receiving bone marrow and organ transplants, patients with a severe B-cell lymphoproliferative disorder (BLPD) underwent the first clinical trial examining CD24-inhibiting drugs [5, 56]. Two mAbs were administered to the patients: ALB9-targeted CD24 and BL13-targeted CD21. The outcomes proved the therapy was well tolerated. Due to CD24 expression on granulocytes, temporary neutropenia occurred in all patients. In seven individuals with monoclonal B-cell proliferation, the therapy was unsuccessful. Sixteen individuals with oligoclonal BLPD, on the other hand, experienced total remission. Of the 16 patients who experienced complete remission, two experienced relapses due to persistent immunodeficiency brought on by graft (marrow) rejection and acute GVHD, respectively. The patient with GVHD eventually passed away. After an average follow-up of 35 months, 11 individuals were still alive and disease-free [56].

In a separate open multicenter study, 58 patients with aggressive BLPD that had occurred after bone marrow transplantation (BMT) or organ transplantation were given specific anti-CD21 and anti-CD24 murine mAbs for 10 days. By achieving complete remission in 36 of the 59 episodes of BLPD in the 58 patients, this combination therapy demonstrated significant clinical activity (61%). Low recurrence rates (3 of 36, 8%) were observed. 46% of patients survived over the long-term overall (median follow-up: 61 months. The only other factor that made a substantial difference to the poor survival was the tumor burden. Anti-CD24 mAb therapy therefore seems to be a rather secure and successful treatment for severe posttransplant BLPD [5]. However, more clinical studies are required to confirm this tactic.

The DCs cytokine-induced killer (CIK) cells loaded with the CD24 peptide (DC/CIK-CD24) were utilized as immunotherapies for primary HCC patients who underwent radical resection in another single-arm, single-institution phase I/II clinical research [57]. After radical resection, two or four rounds of DC/CIK immunotherapy were given to each of the 36 patients with primary HCC. The survival rates of patients aged 1–4 years were assessed during the follow-up. The medication was safe, with transitory fever (grade 3), which occurred in 19% of patients during the trial, being the most frequent side effect. No adverse events of grade 3 or higher were reported [57]. Patients who received trial treatment twice and four times had 4-year overall survival rates of 47% and 53%, respectively. The patients' regulatory T cells reduced compared to baseline after receiving the DC/CIK-CD24 autotransfusion, although CD3^+^, CD4^+^, and CD56^+^ marginally increased [57].

There are further clinical trials testing CD24Fcin patients with advanced solid tumors (NCT04552704) and melanoma (NCT04060407). These trials were phase I/II studies to determine the safety and tolerability of the CD24 extracellular domain-IgG1 Fc domain recombinant fusion protein, CD24Fc (CD24Fc), in patients with advanced solid tumors who developed debilitating immune-related adverse events (irAEs) from immune checkpoint inhibitors (ICIs) and to determine if CD24Fc shortens the recovery time of irAEs and increases the recovery rate of irAEs in cancer patients with grade 2 or 3 irAEs. Three out of the six patients who entered in the phase I study of one of these trials (NCT04552704) had already been terminated early by the sponsor, most likely as a result of the serious adverse effects. Before recruiting ever began, another trial (NCT04060407) was withdrawn. In this phase I/II clinical trial, patients without prior exposure to anti-PD1/L1 checkpoint inhibitors were examined for safety and efficacy of CD24Fc in combination with ipilimumab and nivolumab. Table 1 provides an overview of all relevant CD24-targeting drug clinical trials.

**Table 1:** Clinical trials of targeting CD24

NCT ID	Type of disease or condition	Phase	Agent	Primary outcomes	Enrollment	Status	Results
NCT04552704	Advanced solid tumors	I/II	CD24 agonist CD24 Extracellular Domain-IgG1 Fc Domain Recombinant Fusion Protein CD24Fc	Safety, tolerability, recovery from irAEs	3	Terminated	Yes
NCT04060407	Melanoma	Ib/II	CD24 agonist, CD24Fc Drug: Ipilimumab Drug: Nivolumab	Safety, tolerability	0	Withdrawn	No
N/A	BLPD	I/II	ALB9, BL13	Safety, tolerability	58	Completed	Yes
N/A	Resected HCC	I/II	CD24-loaded DC/CIK autotransfusion	Safety, efficacy	36	Completed	Yes
NCT03960541	HIV infections dyslipidemias	II	Efprezimod Alfa (CD24Fc, MK-7110)	Safety, AEs, tolerability	8	Terminated	Yes
NCT04747574	SARS-CoV-2	I	EXO-CD24	AEs	35	Unknown	No
NCT04317040	Coronavirus disease 2019 (COVID-19)	III	Efprezimod alfa Placebo	Time to improvement, AEs, mortality,	234	Completed	Yes
NCT01214512	Colorectal cancer	N/A	Blood-sample based diagnostic assay	Colonoscopy and CD24 assay correlation	229	Completed	No
NCT04902183	Covid19	II	CovenD24, Exosomes Overexpressing CD24	AEs, Improvement in COVID-19 status from severe to moderate	90	Recruiting	No
NCT04095858	aGVHD in AML with HSCT	III	Efprezimod alfa Placebo Methotrexate Tacrolimus	180-Day Grade III-IV Acute GVHD-Free Survival (aGFS) Overall Survival (OS) Disease-Free Survival (DFS)	11	Terminated	No
NCT02650895	Healthy volunteers	I	Efprezimod alfa Saline	Safety, AEs, Cmax of CD24Fc	40	Completed	Yes
NCT04969172	COVID-19 Disease	II	Exosomes overexpressing CD24	Safety of EXO-CD24, clinical improvement of COVID-19 disease	155	Active, not recruiting	No
NCT02663622	aGVHD following myeloablative allogeneic HSCT	II	Efprezimod alfa Methotrexate Tacrolimus Placebo	AEs, Safety	44	Completed	Yes
NCT01265225	Breast cancer	N/A	N/A	Prognostic Value of Stem Cell Related Markers	0	Withdrawn	No
NCT04907422	Carcinoma Ex pleomorphic adenoma of salivary glands	N/A	N/A	Diagnostic and prognostic accuracy of gold nanoparticles in salivary gland tumors	60	Completed	No

AEs, adverse events; AML, acute myeloid leukemia; irAEs, immune-related adverse events; EXO, exosomes; HSCT, hematopoietic stem cell transplantation; HCC, hepatocellular carcinoma; BLPD, B-cell lymphoproliferative disorder; aGVHD, acute graft-versus-host disease; N/A, not available; OS, overall survival; DFS, disease-free survival

Additionally, administration of soluble CD24 reduces systemic immunopathology linked to COVID-19. By binding to extracellular high-mobility group box 1 and heat shock proteins, soluble CD24 (CD24Fc) inhibits the DAMP-induced broad inflammatory response. It also controls the Siglec10-Src homology 2-containing phosphatase 1 pathway, which is a downstream signaling pathway. Clinical effectiveness results from a recent randomized phase III trial (NCT04317040) assessing CD24Fc in individuals with severe COVID-19 were positive. This was the only institution in the SAC-COVID study to analyze the effect of CD24Fc therapy on immunological homeostasis in COVID-19 patients. The clinical characteristics of the CD24Fc vs. placebo groups were matched, and 22 patients were enrolled. The findings demonstrated systemic hyper-activation of several cellular compartments, including CD8^+^ T cells, CD4^+^ T cells, and CD56^+^ NK cells, in individuals with severe COVID-19. Treatment with CD24Fc reduced this systemic inflammatory response, causing NK and T cells to revert to equilibrium without affecting the anti-spike protein antibody response. The systemic cytokine response was markedly reduced by CD24Fc, as were the cytokine coexpression and network connection associated with the severity and etiology of COVID-19. The data support further research into CD24Fc as a new therapy against severe COVID-19 since it can quickly reduce systemic inflammation and restore immunological homeostasis in people with SARS-CoV-2 infection [58].

### Challenges for CD24/Siglec-10 target therapy

Due to CD24's expression in immune and nervous system cells as well as cancer cells, cross-reactivity poses the biggest threat to cancer immunotherapy. Immunosuppression and cognitive dysfunction consistent with inhibition of immune cell growth [21, 59–61] and neurogenesis [62–64] are the side effects of mAb therapy that are most likely to occur. The absence of binding of the anti-CD24 mAb to human red blood cells suggests that it does not result in hemolytic anemia in humans [29]. However, because these anti-human CD24 mAbs do not cross-react with murine CD24, the outcomes of preclinical research employing mice models might not be sufficient to fully comprehend any potential adverse consequences of this therapy in humans.

The fast homeostatic proliferation of T cells brought on by a deficiency of CD24 on DCs can kill immunocompetent mice. Anti-CD24 mAb's off-target destruction of DCs is therefore expected to cause an increase of T cells that will be extremely harmful to the human host [65]. A significant decrease in the number of circulating B cells can also be caused by the anti-CD24 mAb by killing growing B cells. Additionally, anti-CD24 mAb stops T cells from being co-stimulated to further promote immunosuppression [66, 67].

### Future directions

Although it has been thought that cancer immunotherapy that targets the CD24/Siglec-10 signal pathway is a promising approach, further research is required due to the intricacy of the signaling system and the widespread expression of CD24/Siglec-10 in many normal cell types. As a result of off-target cross-reactions, CD24 glycosylation and its expression variations can make it more challenging to prevent unforeseen negative effects. Despite the fact that numerous anti-CD24 mAbs have been produced, they only bind to the peptide backbone (SWA11 [68] and the carbohydrates BA-1 [69] and SN3b [70]). There is an urgent need for more targeted anti-CD24/Siglec-10 mAbs that can effectively target tumor-specific forms of CD24/Siglec-10 and glycosylation variations. Additionally, CD24 peptides or nanoparticles that are loaded with CD24 could be a cutting-edge method for blocking the CD24/Siglec-10 signaling pathway. Bispecific T-cell engagers (BiTEs) that target CD24/Siglec-10 and additional antigens like CD3 may also be a promising immunotherapeutic approach to pursue, since these molecules can also activate CD24-directed T cells to kill cancer cells [71]. Finally, the use of chimeric antigen receptor T (CAR-T) cells in cancer immunotherapy is another possibility worth considering. In the third generation of CD24-CAR NK cells designed to treat ovarian cancer, it was discovered that both original tumor cells and CD24^+^ ovarian cell lines were specifically eliminated by the CD24-NKCAR cells [72].

## Conclusion

The CD24/Siglec-10 relationship through macrophage antigen presentation has drawn a lot of interest because it can function as a significant innate immunological checkpoint. We hypothesize that anti-CD24 immunotherapy can inhibit the CD24/Siglec-10 signaling pathway and destroy the tumor cell via the following pathways:

1. Anti-CD24 antibodies bind to tumor cells to block the “don’t eat me” signal and directly disrupt the CD24/Siglec-10 signaling pathway.
2. Anti-CD24 antibodies bind to tumor cells and trigger apoptosis in those cells.
3. ADCP is induced on tumor cells by anti-CD24 antibodies that bind to macrophages.
4. Anti-CD24 antibodies bind to NK cells and cause tumor cells to undergo antibody-dependent cellular cytotoxicity (ADCC).
5. By binding to macrophages during antigen presentation processing, anti-CD24 antibodies stimulate T cells to release cytokines that destroy tumor cells.

Targeting the CD24/Signlec-10 signaling pathway is a promising cancer immunotherapy strategy, according to numerous studies, including preclinical studies and clinical trials. However, more research is still needed to fully understand the mechanism of action, including how to reduce or avoid off-target side effects before adequate clinical trial exploration. The development of novel mAbs with high specificity and efficacy against glycosylation variations, CD24 peptides, BiTEs, CAR-T, and/or CD24-CAR NK cells could be among the tactics used in CD24/Siglec-10-targeted cancer immunotherapy in the future.

## Data Availability

The datasets in the current study are from published databases.

## Abbreviations

ADCC: Antibody-dependent cellular cytotoxicity
ADCP: Antibody-dependent cell phagocytosis
BART: Binder of arl two
BiTEs: Bispecific T-cell engagers
BLPD: B cell lymphoproliferative disorder
BMT: Bone marrow transplantation
BRCA: Breast invasive carcinoma
CAR-T: Chimeric antigen receptor T
CD24: Cluster of differentiation 24
CEL: Celastrol
ceRNA: Competitive endogenous RNA
cfDNA: Cell-free DNA
CIK: Cytokine-induced killer
CK4: Cytokeratin 4
CRT: Calreticulin
CSCs: Cancer stem cells
CTCs: Circulating tumor cells
CTLA-4: Cytotoxic T-lymphocyte-associated protein 4
DAMP: Danger-associated molecular pattern
DLBCL: Diffuse large B-cell lymphoma
Evs: Extracellular vesicles
GPI: Glycosylphosphatidylinositol
GVHD: Graft-versus-host disease
HCC: Hepatocellular carcinoma
HDAC: Histone deacetylase
HIF1α: Hypoxia-inducible factor-1alpha
HMGB1: High-mobility group box 1
HSA: Heat-stable antigen
HSCT: Hematopoietic stem cell transplantation
ICIs: Immune checkpoint inhibitors
IgG1: Immunoglobulin G1
irAEs: Immune-related adverse events
ITIMs: Immunoreceptor tyrosine-based inhibition motifs
L1CAM: L1 cell adhesion molecule
L1-CAM: L1-cell adhesion molecule
LAG3: Lymphocyte activating gene 3
mAbs: Monoclonal antibody
MCL: Mantle cell lymphoma
NAFLD: Non-alcoholic fatty liver disease
NCAM: Neural cell adhesion molecule
NSCLC: Non-small cell lung cancer
OC: Ovarian cancer
PARP1: Poly(ADP-ribose) polymerase 1
PD-1/PD-L1: Programmed death-1/programmed death ligand-1
Siglec-10: Sialic acid-binding immunoglobulin-like lectin-10
SIRPa: Signal regulatory protein α
TAM: Tumor-associated macrophages
TME: Tumor microenvironment
TNBC: Triple-negative breast cancer
